# The protective role of myeloid-derived suppressor cells in concanavalin A-induced hepatic injury

**DOI:** 10.1007/s13238-014-0069-5

**Published:** 2014-07-01

**Authors:** Wenli Diao, Fangfang Jin, Bing Wang, Chen-Yu Zhang, Jiangning Chen, Ke Zen, Limin Li

**Affiliations:** 1 Jiangsu Engineering Research Center for MicroRNA Biology and Biotechnology, State Key Laboratory of Pharmaceutical Biotechnology, School of Life Sciences, Nanjing University, Nanjing, 210093 China; 2 People’s Liberation Army 404 Hospital, Weihai, 264200 China

**Keywords:** myeloid derived suppressor cells, T cell-mediated hepatitis, ROS, glucocorticoids, concanavalin A (ConA), adoptive transfer, glucocorticoid treatment

## Abstract

The mechanism underlying T cell-mediated fulminant hepatitis is not fully understood. In this study, we investigated whether myeloid derived suppressor cells (MDSCs) could prevent the concanavalin A (ConA)-induced hepatitis through suppressing T cell proliferation. We observed an increase in the frequencies of MDSCs in mouse spleen and liver at early stage of ConA treatment, implicating that the MDSCs might be involved in the initial resistance of mice against ConA-mediated inflammation. Subpopulation analysis showed that the MDSCs in liver of ConA-induced mice were mainly granulocytic MDSCs. Adoptive transfer of the bone marrow-derived MDSCs into ConA-treated mice showed that the MDSCs migrated into the liver and spleen where they suppressed T cell proliferation through ROS pathway. In addition, the frequencies of MDSCs in mice were also significantly increased by the treatment with immune suppressor glucocorticoids. Transfer of MDSCs into the regulatory T cell (Treg)-depleted mice showed that the protective effect of MDSCs on ConA-induced hepatitis is Treg-independent. In conclusion, our results demonstrate that MDSCs possess a direct protective role in T cell-mediated hepatitis, and increasing the frequency of MDSCs by either adoptive transfer or glucocorticoid treatment represents a potential cell-based therapeutic strategy for the acute inflammatory disease.

## INTRODUCTION

T cell-mediated immune response plays a central role in inducing hepatocellular injury during hepatitis. Activated T cells are detected in a variety of human liver diseases including autoimmune hepatitis, chronic active hepatitis B or C, alcoholic liver diseases, hepatitis ischemia/reperfusion injury, and allograft rejection (Hong et al., 2002; Lafdil et al., 2009). T cell-mediated hepatitis can be induced in rodents by injection of concanavalin A (ConA): a lectin, originally extracted from Canavalia brasiliensis plant, which rapidly induces clinical and histological hepatitis, including up regulation of transaminase activity and CD4^+^CD69^+^ T cells and down regulation of NK1.1^+^ and CD3^+^ NKT cells within 24 h (Hines et al., 2007). In the past two decades, major progress has been made in understanding of the molecular and cellular mechanisms underlying T cell-mediated liver injury through use of this model. Evidence suggests that ConA-induced T cell-mediated hepatitis is initiated and tightly controlled by interactions between multiple cell types and cytokines. Immune cells involved in ConA-induced hepatitis include CD4^+^ T cells, natural killer T cells, Tregs, Kupffer cells (Erhardt et al., 2007), neutrophils, and eosinophils (Lafdil et al., 2009). The inflammatory cytokines, IFN-γ, IL-4 (Jaruga et al., 2003), and TNF-α (Wolf et al., 2001) have been shown to play an essential role in T cell-mediated hepatitis. Additionally, IL-2, IL-6, IL-10, and IL-22 are also involved in ConA-induced liver injury (Radaeva et al., 2004; Erhardt et al., 2007; Takahashi et al., 2011).

Myeloid-derived suppressor cells (MDSCs) represent a heterogeneous population of immature myeloid cells including myeloid progenitors, macrophage precursors, granulocytes, and dendritic cells, which share a common capacity of suppressing immune responses (Gabrilovich, 2004). Murine MDSCs are characterized by the surface co-expression of Gr-1 and CD11b, and are further subdivided into two major groups: CD11b^+^ Ly6G^+^ Ly6C^low^ granulocytic MDSCs and CD11b^+^ Ly6G^-^ Ly6C^high^ monocytic MDSCs. Granulocytic MDSCs and monocytic MDSCs differ in their abilities to suppress T cell responses (Gabrilovich, 2004). Monocytic MDSCs suppress T cell proliferation by high levels of inducible nitric oxide synthase (iNOS) and Arginase 1 (ARG1), while granulocytic MDSCs mainly through high levels of reactive oxygen species (ROS) and Arginase 1 (ARG1) (Gabrilovich, 2004; Bronte and Zanovello, 2005; Gabrilovich and Nagaraj, 2009; Gabrilovich et al., 2012). MDSCs exploit various mechanisms to influence both innate and adaptive immune responses (Gabrilovich et al., 2012). In short, MDSCs can deprive T cells of L-cysteine and L-arginine which are essential for their growth and differentiation, generate oxidative stress that cause the loss of the TCR ζ-chain, decrease CD62L expression to interfere with T cell migration and viability, and induce the activation and expansion of regulatory T (Treg) cell populations. Under pathological conditions such as tumor growth and graft-versus-host disease, the documented accumulation of MDSCs in patients and mice suggested a critical contribution of MDSCs to these immunosuppressive conditions (Almand et al., 2001). The expansion of MDSC and its protective role in suppressing body inflammation and autoimmunity has also been observed in various pathophysiological conditions. Recent findings suggest that the accumulation of MDSCs may be related to inflammatory bowel disease (Zhang et al., 2013), type 1 diabetes (Yin et al., 2010; Xia et al., 2011), systemic lupus erythematosus, inflammatory eye disease, multiple sclerosis (Cripps and Gorham, 2011), and hepatitis B and C virus infection (Chen et al., 2011; Hegde et al., 2011; Tacke et al., 2012; Cai et al., 2013). Furthermore, Hegde et al. (Hegde et al., 2011) recently observed an upregulation of MDSCs subsequent to cannabidiol treatment of autoimmune liver injury. However, the direct anti-inflammatory role of MDSCs in autoimmune hepatitis remains unclear.

In the present study, we characterized the role of MDSCs in ConA-induced mouse hepatitis by detecting serum cytokines and activation markers of lymphocytes. The tissue localization of adoptive transferred MDSCs in mice under inflammatory or non-inflammatory conditions were determined. The protective effects of MDSCs on experimental hepatitis were further analyzed through increasing the frequency of MDSCs in mice through the adoptive transfer of MDSCs into mice or the glucocorticoid treatment.

## RESULTS

### ConA-induced hepatitis mice model

**Figure 1:**
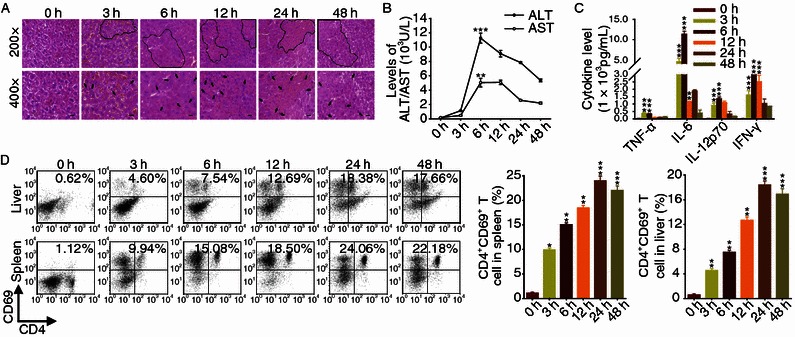
**ConA induced mouse acute hepatitis**. (A) Photomicrographs of representative mouse liver from mice treated with ConA (20 mg/kg) for 0, 3, 6, 12, 24, and 48 h (H&E staining, original magnification 200× and 400×). (B) Serum ALT and AST levels at various time point post-ConA injection. (C) Circulating TNF-α, IL-6, IFN-γ, and IL-12p70 levels at various time point post-ConA injection. (D) ConA-mediated activation of mouse CD4^+^ T cells. At various time point post-ConA injection, hepatic MNC were isolated and CD4^+^CD69^+^ T cells were analyzed. Representative scatter plots are presented (left) and the histogram (right) represents the statistical analysis of the percentages of CD69-positive CD4^+^ T cells. All the values are shown as mean ± SEM. *P < 0.05, **P < 0.01, and ***P < 0.005 (*n* = 5–8)

In this experiment, 8–10 weeks C57BL/6 mice were injected either with 0.9% NaCl or 20 mg/kg ConA intravenously. Following ConA administration, mice were sacrificed at different time points. Multiple evidences collectively indicated that the ConA-induced hepatitis mice model was successfully developed. H&E staining indicated recipients of ConA had increasing liver necrosis compared to the control (Fig. 1A). Examination of serum ALT and AST activities (Fig. 1B) revealed that the ConA treatment caused a severe liver injury. The peak of ALT and AST activities was observed at the time point of 6 h. The enhanced hepatitis injury over time was also reflected by the up regulation of serum TNF-α, IL-6, IL-12p70, and IFN-γ (Fig. 1C), with the peak concentration at the time point of 6 h, followed by a rapid decrease within 48 h. In addition, splenic and hepatic T lymphocytes were rapidly activated as demonstrated by the expression of CD69^+^ on CD4^+^ lymphocytes with a time dependent manner (Fig. 1D). Together, the data demonstrate that the persistence of liver injury, T cell activation, and the peak of ALT, AST, cytokines levels was 6 h after ConA injection, which was the time point chosen for the next experiment.

### CD11b^+^Gr-1^+^ cells are expanded during acute T cell-mediated hepatitis

**Figure 2:**
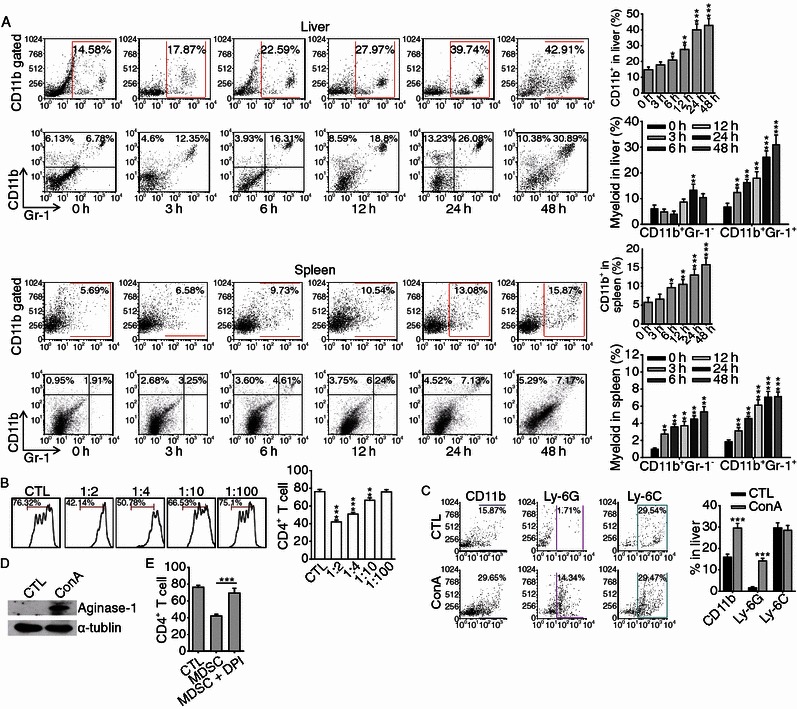
**Mouse MDSCs were expanded and activated following ConA treatment**. (A) Analysis of CD11b^+^ cells, CD11b^+^ Gr-1^-^ cells, and CD11b^+^ Gr-1^+^ MDSCs in ConA-treated mouse liver and spleen. Representative scatter plots (left) are presented and the histogram (right) represents the statistical analysis of the percentages of CD11b^+^ cells, CD11b^+^ Gr-1^-^ cells, and CD11b^+^ Gr-1^+^ MDSCs. (B) Inhibition of mouse liver MDSCs on T cell proliferation. MDSCs were isolated from ConA-treated mice and the inhibition assay was performed at various MDSC vs. T cell ratio. The histogram (lower panel) represented the statistical analysis of CD4^+^ T cell proliferation. (C) Analysis of CD11b^+^Ly6G^-^Ly6C^high^ monocytic MDSCs and CD11b^+^Ly6G^+^Ly6C^low^ granulocytic MDSCs from the ConA-treated mouse liver. Normal mice were used as control (CTL). (D) Protein level of Arginase-1 were detected in MDSCs of ConA-induced mice liver. (E) Inhibition of T cell proliferation by MDSCs isolated from the liver of ConA-treated mice at the ratio of 1:2 (MDSC vs. T cell) with or without DPI. All the values are shown as mean ± SEM. *P < 0.05, **P < 0.01, and ***P < 0.005 (*n* = 5–8)

MDSCs play an important role during benign inflammation *in vivo*, shaping immune responses to viral antigens, influencing antibody production, and down regulating T cell responses to auto-antigens (Haverkamp et al., 2011). To examine the potential role of MDSC in T cell-mediated hepatitis, the levels of CD11b^+^ cells, CD11b^+^Gr-1^−^ cells, CD11b^+^Gr-1^+^ MDSCs (Fig. 2A) were analyzed in the spleen and liver over time. As shown in Fig. 2A, ConA injection rapidly induced accumulation of CD11b^+^ cells in the liver, and the majority of these cells are CD11b^+^Gr-1^+^ MDSCs. To characterize the suppressive capacity of CD11b^+^Gr-1^+^ MDSCs from inflammatory liver, we purified MDSCs from the inflammatory liver and co-cultured them with CFSE-labeled T cells at different ratios. As shown in Fig. 2B, MDSCs purified from ConA-treated mouse liver strongly suppressed T cell proliferation. Subpopulation analysis showed that the accumulated CD11b^+^Gr-1^+^ MDSCs are mainly from the granulocytic subsets (Fig. 2C). Further, we sorted MDSC from the liver of mice treated with ConA, and found that Arginase-1 was enriched in the isolated cells (Fig. 2D). To determine whether MDSCs inhibit T cell proliferation through ROS, we used DPI as ROS inhibitor and found that DPI could significantly inhibit the function of MDSCs isolated from the ConA-treated mouse liver (Fig. 2E). These data suggest that MDSCs may regulate T cell function during ConA-induced mouse hepatitis through ROS pathway.

### BM-MDSCs migrate to lymphocytes-accumulated organs and exert a protective effect on ConA-induced hepatitis

**Figure 3:**
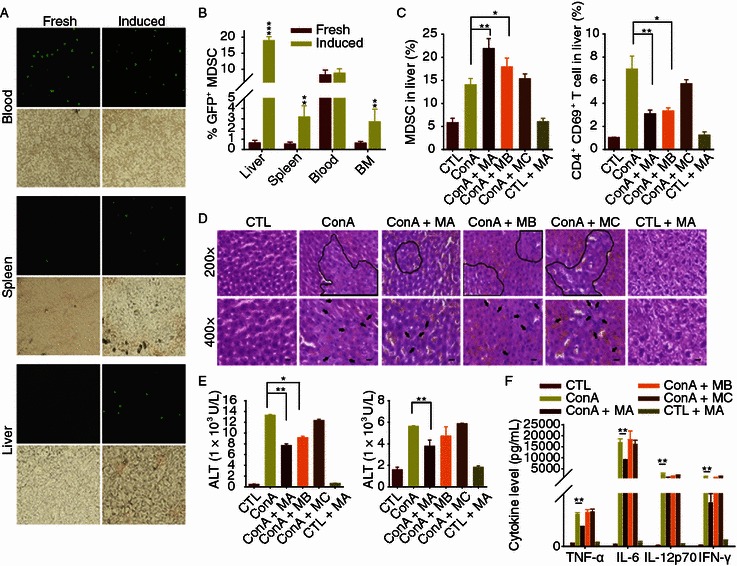
**Adoptively transferred MDSCs attenuated ConA-induced mouse hepatitis**. (A) Tracing of adoptively transferred MDSCs in mice with or without ConA (20 mg/kg) treatment. GFP-labeled BM-MDSCs were prepared from GFP transgenic mice and then injected into C57BL/6 mice intravenously. At the same time, mice were treated with or without ConA. (B) Analysis of the percentages of GFP-labeled BM-MDSCs in ConA-treated mouse liver, spleen, blood, and BM. (C) Frequencies of MDSCs and CD4^+^ CD69^+^ T cells in mouse liver. Different concentrations of BM-MDSCs (MA, MB, and MC represented 5 × 10^6^, 5 × 10^5^, and 5 × 10^4^ cells of MDSCs, respectively, the same as in panels D–F) were transferred intravenously to mice with ConA treatment. (D) Photomicrographs of representative liver sections from mice treated with ConA and MDSCs (H&E staining, original magnification, 200× and 400×). (E) Serum levels of ALT and AST in mice treated with ConA and BM-MDSCs. (F) Levels of circulating TNF-α, IL-6, IFN-γ, and IL-12p70 in mice treated with ConA and BM-MDSCs. Values are shown as mean ± SEM. *P < 0.05, **P < 0.01 and ***P < 0.005 (*n* = 5–8)

To directly view the effect of transferred BM-MDSCs on attenuation of ConA-induced hepatitis in mouse model, we isolated BM from GFP transgenic mice, induced BM-MDSCs with GM-CSF and IL-6, and traced their localization in mice with ConA-induced hepatitis. Mice without ConA treatment were used as control. As shown in Fig. 3A and 3B, at only 3 h after ConA injection, GFP^+^ BM-MDSCs were readily detected in BM, spleen, and liver tissues, but not visible in BM, spleen, and liver of control mice, suggesting the homing of exogenous MDSCs is modulated by inflammatory condition. Furthermore, as shown in Fig. 3C, the transferred BM-MDSCs significantly increased the frequency of CD11b^+^Gr-1^+^ MDSCs in mouse liver. Adoptive transfer of BM-MDSCs also markedly decreased the population of CD4^+^CD69^+^ T cells. These results suggest that the transferred BM-MDSCs could migrate to certain organs where lymphocytes accumulated under inflammatory condition.

To better understand the effect of MDSCs on the suppression of T cell-mediated hepatitis development, we compared ConA-induced hepatitis with or without exogenous transfer of BM-MDSCs in different number. Examination of liver pathology showed massive necrosis in mice without BM-MDSCs transfer, but not in mice transferred with BM-MDSCs (Fig. 3D). The levels of AST and ALT were dramatically decreased in serum of mice transferred with BM-MDSCs (Fig. 3E). Since TNF-α, IL-6, IL-12p70, and IFN-γ have been shown to play an important role in T cell-mediated hepatitis (Lafdil et al., 2009), we compared the serum TNF-α, IL-6, IL-12p70, and IFN-γ levels in mice with or without BM-MDSCs transfer. As expected, the ConA-induced elevation of TNF-α, IL-6, IL-12p70, and IFN-γ levels significantly attenuated in BM-MDSCs-transferred mice compared to those of non BM-MDSCs-transferred mice (Fig. 3F). From the concentration gradient we could also find that the effect of BM-MDSCs on protection of ConA-induced injury was dose-dependent. When the number of MDSC was less than 5 × 10^5^, the protection effect was disappeared in ConA-induced mice. Besides, there was also no significant difference of liver damage, level of ALT/AST and cytokine between only MDSC and vehicle control, suggesting that MDSCs themselves do not damage liver cells.

### Glucocorticoids protect mice from ConA-induced hepatitis through expanding MDSC

**Figure 4:**
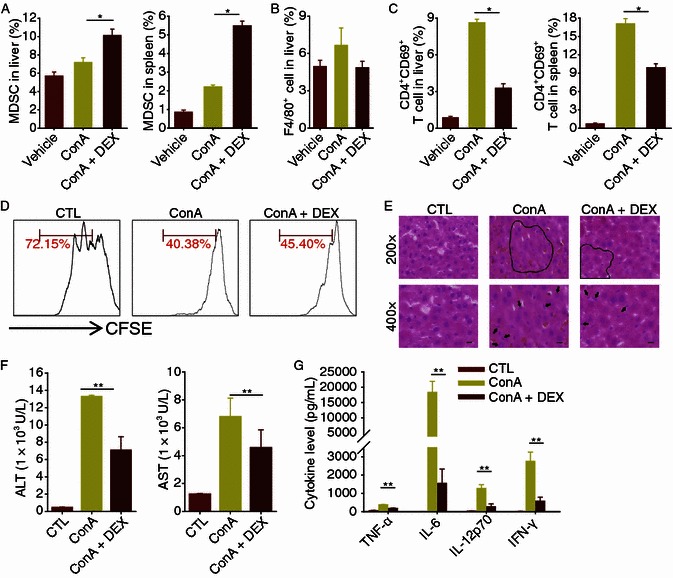
**Dexamethasone treatment protected mice from ConA-induced hepatitis through expanding MDSCs**. (A) The levels of MDSCs in liver and spleen of ConA-treated mice that were injected with or without DEX. Mice were injected intraperitoneally with dexamethasone (DEX, 1 mg/kg bodyweight) and ConA (20 mg/kg). (B) The levels of F4/80^+^ macrophages in liver of ConA-treated mice that were injected with or without DEX. (C) The levels of CD4^+^CD69^+^ T cells in liver and spleen of ConA-induced mice that were injected with or without DEX. (D) The inhibition of T cell proliferation by MDSCs isolated from mouse livers that were treated with or without DEX. (E) The representative H&E staining of liver sections of ConA-treated mice that were injected with or without DEX. (F) Serum ALT and AST levels in ConA-treated mice that were injected with or without DEX. (G) Circulating TNF-α, IL-6, IFN-γ, and IL-12p70 levels in ConA-treated mice that were injected with or without DEX. Values are shown as mean ± SEM. *P < 0.05, **P < 0.01, and ***P < 0.005 (*n* = 5–8)

Glucocorticoids (GC) provide the most effective anti-inflammatory treatments for many inflammatory and immune diseases, including asthma, rheumatoid arthritis, inflammatory bowel disease, and autoimmune diseases (Barnes and Adcock, 2009). Systemically active conventional corticosteroids have played a significant role in the induction of remission in autoimmune liver diseases, liver transplantation, and virus induced hepatitis (Ducci and Katz, 1952; Czaja et al., 1984; Fujiwara et al., 2010). Recent study by Varga et al. (Varga et al., 2008) suggested that the GC treatment might induce an activated, anti-inflammatory monocyte subset in mice that resembles myeloid-derived suppressor cells. In the present study, we treated mice with synthetic GC such as dexamethasone (DEX) after the process of ConA-induced experimental hepatitis. The results showed that DEX treatment strongly increased the frequency of CD11b^+^Gr-1^+^ MDSCs in mice spleen and liver (Fig. 4A). Accordingly, the populations of CD4^+^ CD69^+^ T cells in mouse spleen and liver were strongly down regulated by DEX treatment (Fig. 4C). MDSCs isolated from liver of mice treated with DEX also have the ability to inhibit T cell proliferation *in vitro* (Fig. 4D). Importantly, there is no significantly difference of macrophages between the group of vehicle, ConA, ConA and DEX (Fig. 4B), suggesting that CD11b^+^Gr-1^+^ MDSCs are the main functional cells induced by DEX.

Next, we compared the ConA-induced mouse hepatitis with or without DEX treatment. As shown in Fig. 4E–G, DEX treatment strongly attenuated the ConA-induced hepatitis in mice, accompanied with fewer necrotic liver cells (Fig. 4E), lower activities of ALT and AST (Fig. 4F). Compared to mice treated with ConA alone, mice treated with ConA and DEX also displayed a significant lower level of serum TNF-α, IL-6, IL-12p70, and IFN-γ (Fig. 4G). These results implicate that the protection of DEX against ConA-induced hepatitis may be dependent on the induction of MDSCs.

### MDSCs protect ConA-induced mice hepatitis independent of Tregs

**Figure 5:**
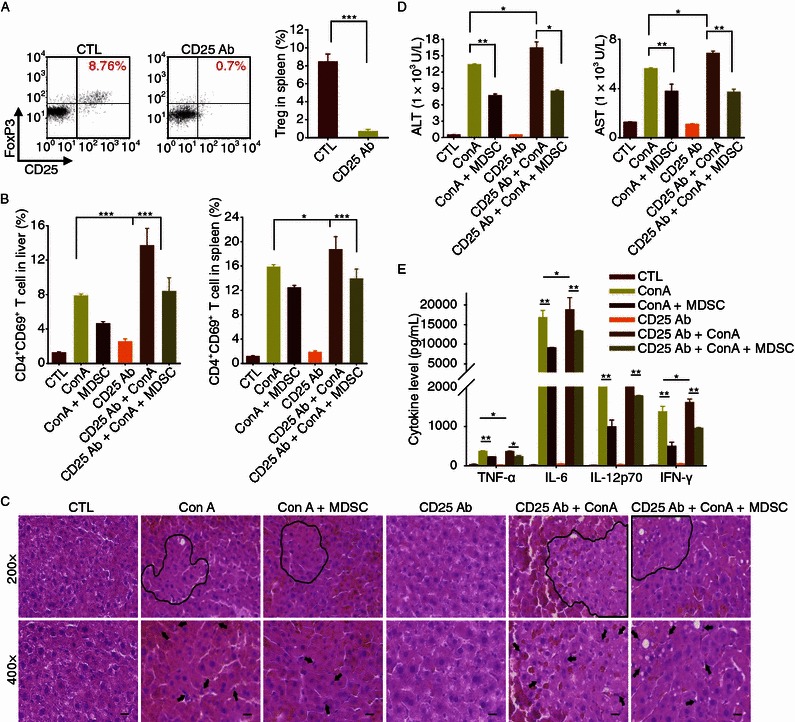
**MDSCs protected ConA-induced mice hepatitis in a Treg-independent manner**. (A) Depletion of Tregs in mouse spleen by treating mice with rat anti-mouse CD25 antibody (CD25 Ab) (left). The histogram (right) represented the statistical analysis of the percentages of Tregs in spleen. (B) Analysis of the effect of Treg depletion on ConA-treated mice. The percentages of CD4^+^CD69^+^ T cells in liver and spleen were presented. (C) Photomicrographs of representative H&E staining of mouse liver sections 6 h post-ConA injection (original magnification, 200× and 400×). During ConA treatment, control and Treg-depleted mice were transferred with or without BM-MDSCs. (D) Serum levels of ALT and AST in ConA-treated control or Treg-depleted mice with or without adoptive transfer of BM-MDSCs. (E) Levels of circulating TNF-α, IL-6, IFN-γ, and IL-12p70 in ConA-treated control or Treg-depleted mice with or without adoptive transfer of BM-MDSCs. Values are presented as mean ± SEM. *P < 0.05, **P < 0.01, and ***P < 0.005 (*n* = 5–8)

Tregs have been reported as one of the cells targeted by MDSCs (Pan et al., 2008). To investigate the relationship between MDSCs and Tregs in our model, we transiently depleted Tregs by injecting rat anti-mouse CD25 antibody (Yu et al., 2010). As shown in Fig. 5A, Tregs in mouse spleen were effectively depleted by CD25 antibody. Constructing ConA-induced hepatitis model on Treg-depleted mice, we found that depletion of Tregs upregulated CD4^+^CD69^+^ T cells in the liver and spleen (Fig. 5B). Furthermore, after transferring the BM-MDSCs into Treg-depleted mice which were also treated with ConA, we found that exogenous BM-MDSCs significantly down regulated the CD4^+^CD69^+^ T cells in the liver and spleen. Examination of mouse liver tissues showed severer necrosis in Treg-depleted mice than mice without depletion of Tregs. As shown in Fig. 5C, transfer of BM-MDSCs could alleviate liver injury significantly. As expected, the levels of AST and ALT (Fig. 5D) or TNF-α, IL-6, IL-12p70, and IFN-γ (Fig. 5E) were dramatically decreased in the serum of mice with BM-MDSCs transfer compared to those of mice without BM-MDSCs transfer. These results implicate that MDSC can protect mouse liver from ConA-mediated injury in a Treg-independent manner.

## DISCUSSION

T cell activation in HCV, HBV, drug intoxication, and alcoholic liver diseases mediated hepatitis has been shown to play a central role in hepatocellular injury. For example, in chronic HBV and HCV infection, although the viruses themselves are not cytopathogenic, activated CD8^+^ T cells kill viral infected hepatocytes, while activation of CD4^+^ T cells produces inflammatory cytokines and intern controls CD8^+^ T cell cytotoxicity contributing to the progression of liver disease (Rehermann, 2000; Rosen et al., 2002; Chang, 2003). It has been well documented that the T cell-mediated hepatitis is controlled by the interactions between cytokines and multiple cells (Tiegs, 2007). Previous studies have shown that MDSCs may be involved in down regulation of immune responses through inhibiting T cell not only in tumor situation, but also in a variety of allogeneic transplant models, autoimmune diseases, and other inflammatory diseases (Garcia et al., 2010; Yin et al., 2010). Chou and co-workers (Chou et al., 2011) discovered that hepatic stellate cells can promote the generation of MDSCs with significant immune inhibitory activity *in vitro* and *in vivo*, suggesting a great clinical application potential of MDSC.

The immune suppressor activity of MDSC has been associated with high Arginase-1 and iNOS activity (Greten et al., 2011). Both Arginase-1 and iNOS are highly expressed in MDSCs of tumor bearing mice. iNOS generates nitric oxide (NO) to suppress T cell function via utilizing L-arginine, while Arginase-1 leads to CD3 ζ-chain downregulation and cell cylce arrest through upregulating the expression of cyclin D3 and cdk4 (Rodriguez et al., 2007; Gabrilovich and Nagaraj, 2009). It is reported that novel mechanism of T cell tolerance is associated with reactive oxygen species (ROS) and peroxynitrite (Nagaraj et al., 2007). Tacke and co-workers (Tacke et al., 2012) reported that hepatitis C virus could induce myeloid suppressor cells to suppress T-Cell responses through the production of ROS. Cai et al. (Cai et al., 2013) revealed that a significant correlation between MDSC levels and HCV disease progression, and MDSCs could suppress T cell function in an Arginase-1-dependent manner. Ilkovitch et al. (Ilkovitch and Lopez, 2009) first confirmed that splenic MDSCs isolated from tumor bearing mice can migrate to and accumulate in the liver, suggesting that tumor induced MDSCs may play an immunosuppressive role in the liver. In the present study, using an established model of ConA-induced hepatitis, we demonstrate that the MDSCs are involved in T cell-mediated liver injury and that MDSC-mediated suppression of early CD4^+^CD69^+^ T cells proliferation can protect mice from ConA-induced hepatitis through ROS pathway.

Glucocorticoids have been the most common immunosuppressants used in the treatment of T cell-mediated liver disease (Suda et al., 2003). The role of these agents in liver diseases and liver transplantation has been well documented. Previous studies have reported that the use of glucocorticoids in the early period of severe hepatitis may prevent the necrosis of liver cells and provide a possibility of liver regeneration (Czaja et al., 1984). Recently, the use of glucocorticoids to treat HBV-related liver failure has become much safer because of the new generation of nucleoside analogs, proton pump inhibitors, and effective infection control measures. Although glucocorticoids have long been used to treat the patients with liver dysfunction, the mechanism underlying the profound effect of glucocorticoids on inflammation remains incompletely understood. Previous studies suggested that the glucocorticoids served in multiple capacities by decreasing inflammation and suppressing immune function by interfering with the function of T lymphocytes, reducing the recruitment of monocytes and macrophages, inhibiting the function of immune competent cells, and inhibiting the release of inflammatory cytokines (Elenkov and Chrousos, 1999; Franchimont et al., 2000; Schleimer et al., 2009). By studying the subpopulation of steroid-resistant CD4^+^ T cells, Lee et al. (Lee et al., 2007) showed that the steroid resistance of T cells was associated with CD25 expression and CD4^+^CD25^int^ cells, which were highly resistance to DEX treatment. In the present study, we demonstrated that the DEX could directly promote the generation of MDSCs from bone marrow cells. Increasing the frequency of MDSCs by glucocorticoids might serve as a novel mechanism underlying the suppressive effect of glucocorticoids on various chronic inflammatory and autoimmune diseases.

In summary, our study demonstrates for the first time that MDSCs, derived from adoptively transfer or directly induction by glucocorticoids treatment, can effectively protect mice from ConA-induced hepatitis through downregulating early T cell proliferation and inflammatory responses. Our finding also provides a novel MDSC-based therapeutic strategy in controlling T cell-mediated hepatitis.

## Materials And Methods

### Reagents

Concanavalin A (ConA), dexamethasone, and DPI were purchased from Sigma-Aldrich (St. Louis, MO). Murine IL-6 and GM-CSF cytokines were from PEPROTECH (Rocky Hill, NJ). PerCP/Cy5.5-conjugated anti-mouse (Gr-1), APC-conjugated anti-mouse CD3, and PE-conjugated anti-mouse CD11b were purchased from Biolegend (San Diego, CA). FITC-conjugated rat anti-mouse CD4, APC-conjugated anti-mouse CD69, and mouse regulatory T cell Staining Kit were purchased from eBioscience (San Diego, CA). Purified NA/LE rat anti-mouse CD25 was purchased from BD Pharmingen. Collagenase Type II and DNase I were purchased from Gibco by life technologies (Carlsbad, CA). Rabbit polyclonal antibody to Arginase-1 was purchased from Cell Signaling Technology (Danvers, MA) and mouse monoclonal antibody to alpha tubulin was purchased from Abcam (Cambridge, MA).

### Animals

Eight-week-old male C57BL/6 mice were obtained from the Nanjing University Animal Center (Nanjing, China). Green fluorescent protein (GFP) transgenic mice were purchased from the Jackson Laboratory (Bar Harbor, ME). All Animal maintenance and experimental procedures were carried out in accordance with the US National Institute of Health Guidelines for Use of Experimental Animals and approved by the Animal Care Committee of the Nanjing University.

### Adoptive transfer of BM-MDSC and dexamethasone treatment

The BM-derived MDSCs were obtained as described previously (Bronte and Zanovello, 2005). Briefly, the cells were planted into the dishes using RPMI 1640 medium supplemented with 2 mmol/L L-glutamine, 10 mmol/L HEPES, 20 µmol/L 2-mercaptoethanol, 150 U/mL streptomycin, 200 U/mL penicillin, 10% FBS and stimulated with combinations of GM-CSF (40 ng/mL) and IL-6 (40 ng/mL). Cells were cultured at 37°C in 5% CO_2_-humidified atmosphere for 4 days. 5 × 10^6^ BM-MDSCs were injected intravenously accompanied ConA injection. In separate experiment, dexamethasone (1 mg per kg bodyweight) was injected intraperitoneally accompanied ConA injection.

### Depletion of Tregs

To deplete Tregs, mice were injected intraperitoneally 0.5 mg purified rat anti-mouse CD25 or isotype control, according to the method of Yu et al. (Yu et al., 2010).

### Isolation of liver MNC and spleen cell preparation

Liver mononuclear cells (MNC) were isolated and purified by the method of Richman et al. (Richman et al., 1979), combined with Percoll density separation (Li et al., 2009). Briefly, livers were mechanically disrupted into 1 mm^3^ pieces and digested for 45 min at 37°C with 0.05% collagenase Type II and 0.001% DNase I in RPMI 1640 medium. After filtered with 70 μm nylon cell strainer (BD Falcon) and centrifuged for 5 min at 50× *g*. Supernatants were centrifuged for 5 min at 400× *g*, pellet was washed with HBSS without Ca^2+^ and Mg^2+^. MNC resuspended in 40% Percoll, were gently overlayed onto 70% Percoll and centrifuged for 20 min at 750× *g*. Purified MNC were collected from the interface for further analysis of hepatic MDSCs and T cells. Spleens were collected in sterile HBSS without Ca^2+^ and Mg^2+^, grinded, and filtered. After erythrocytes were depleted, purified splenic cells were collected for further flow analysis.

### Flow cytometric analysis

Flow cytometry was conducted using BD FACScalibur device and analyzed with FCS express V3. After washing with Hank’s buffer devoid of Ca^2+^ and Mg^2+^ (HBSS), 5 × 10^5^ cells from liver and spleen were blocked using 1% BSA at 4°C for 30 min. CD4, CD69, CD11b, and Gr-1 antibodies were added for incubation in another 30 min at 4°C. Tregs were analyzed by using mouse regulatory T cell Staining Kit (eBioscience).

### T cell proliferation

In order to obtain high purity MDSCs from liver, cell isolation kit (Miltenyi Biotec., Bergisch Gladbach, Germany) was used according to manufacturer’s instructions. For T cell proliferation assay, the splenocytes were firstly separated with lymphocyte separation medium. Lymphocytes were labeled with CFSE according to manufacturer’s instructions (Invitrogen). CFSE-labeled lymphocytes were stimulated with ConA, and lymphocytes were co-cultured at 2:1, 4:1, 10:1, or 100:1 ratio with induced liver MDSCs in 96-well flat bottom plates. For MDSC inhibition assay, an inhibitor of NADPH oxidase, diphenyliodonium chloride (DPI, Sigma-Aldrich) was used as previously reported (Cheng et al., 2013). In brief, 10 μmol/L DPI was used in co-cultured MDSC and lymphocytes. After 4 days, cells were stained with CD3 antibody, and CFSE signal of gated lymphocytes was analyzed. The extent of cell proliferation was quantified by the FCS expressing V3.

### Western blot analysis

MDSCs derived from mice livers were lysed in lysis buffer containing 100 mmol/L Tris (pH 7.5), 150 mmol/L NaCl, 1% Triton X-100, protease inhibitor cocktail of PI and PMSF. The antigens were visualized using the ECL plus detection system (Amersham Pharmacia Biotech). Normalization was performed by blotting the same samples with the anti-alpha tubulin antibody.

### Assay for serum aminotransferase activity

Mice were sacrificed after ConA injection for different time and blood samples were collected in 1.5 mL tubes. After centrifugation at 2,500 rpm for 15 min, serum was recovered and immediately frozen at -70°C. Serum alanine aminotransferase (ALT and AST) activities were determined by using the serum aminotransferase test kit (Nanjing Jiancheng Bioengineering Institute, China) according to the manufacturer’s instructions.

### Measurement of serum cytokine levels

Whole blood was collected without anticoagulant and the serum was isolated by centrifugation. Serum levels of TNF-α, IFN-γ, IL-6, and IL-12p70 were determined using ELISA kits (R&D) according to the manufacturer’s instruction. The absorbance was measured with a wavelength correction (A450 nm) with a microplate reader (Bio-Rad).

### Histopathology

Livers from individual mice were cut into 2 × 4 × 4 mm^3^ sections, fixed in 4% paraformldehyde and embedded in paraffin. 5 μm slices were then cut at various depths in the tissue sections, stained with hematoxylin-eosin (H&E) and examined under light microscopy to determine the level of inflammation.

### Tracing of BM-derived MDSCs in mice induced with ConA

Fluorescent BM-MDSCs were obtained from GFP transgenic mice following GM-CSF and IL-6 induction and injected intravenously (5 × 10^6^/per mouse) 1 h before ConA injection. For tracing the location of fluorescently labeled BM-MDSCs, mice were sacrificed 3 h after injection. The fluorescence of blood was analyzed by fluorescence microscope directly. The spleens and livers were removed for frozen section. Then, fluorescent BM-MDSCs were analyzed by fluorescence microscope.

### Statistical analysis

Data shown are presented as mean ± SEM of at least three independent experiments; differences are considered statistically significant at *P* < 0.05 by Student’s *t*-test.

## ABBREVIATIONS

ARG1: Arginase 1
ConA: concanavalin A
DPI: diphenyliodonium chloride
iNOS: inducible nitric oxide synthase
MDSCs: myeloid derived suppressor cells
MNC: liver mononuclear cells
ROS: reactive oxygen species
Treg: regulatory T cell
